# Agreement analysis and associated factors of SARC-F and SARC-CALF in screening of risk sarcopenia in people living with human immunodeficiency virus

**DOI:** 10.1016/j.clinsp.2024.100565

**Published:** 2025-01-03

**Authors:** Lara Cristina Vieira, Jaine Alves Ximenez, Maria Claudia Bernardes Spexoto

**Affiliations:** a Nutrition Course, Faculty of Health Sciences, Federal University of Grande Dourados (UFGD), Dourados, Mato Grosso do Sul, Brazil; b Posgraduate Program in Food, Nutrition and Health, Faculty of Health Sciences, Federal University of Grande Dourados (UFGD), Dourados, Mato Grosso do Sul, Brazil

**Keywords:** Muscle strength, Sarcopenia, HIV, Screening

## Abstract

**Introduction:**

People Living with Human Immunodeficiency Virus (PLHIV) appear to be at a higher risk of developing sarcopenia. Various factors seem to influence the risk of sarcopenia, and its prevalence may differ depending on the screening tool used. This study aimed to (i) Screen the risk of sarcopenia in PLHIV using the SARC-F and SARC—Calf and identify associated factors; (ii) Analyze the agreement between the instruments in PLHIV.

**Methods:**

Cross-sectional study including PLHIV taking antiretroviral therapy. The authors assessed sarcopenia risk using the SARC-F and SARC—Calf tools with ≥4 and ≥11 cutoff points, respectively, and a wide spectrum of variables was analyzed.

**Results:**

Participated 76 patients (44.9 ± 12.7 years). Sarcopenia risk, according to the SARC-F, was 27.6 % and was associated with socioeconomic status (*p* = 0.004), smoking (*p* = 0.001), disease status (*p* < 0.001), opportunistic infections (*p* = 0.001), CD4 T-cell count (*p* < 0.001), Handgrip Strength (HGS) (*p* < 0.001), and Gait Speed (GS) (*p* = 0,001). Using the SARC—Calf, sarcopenia risk was 36.8 % and was associated with work activity (*p* = 0.029), socioeconomic status (*p* = 0.004), smoking (*p* = 0.009), disease status (*p* < 0.001), opportunistic infections (*p* = 0.015), CD4 T-cell count (*p* = 0.002), HGS (*p* = 0.001), Appendicular Skeletal Muscle Mass Index (ASMMI) (*p* = 0.009), and GS (*p* < 0.001). The agreement between tools was moderate (*k* = 0.49).

**Conclusion:**

Sarcopenia risk, as determined by both tools, was higher in low-income PLHIV with opportunistic infections, CD4 T-cell count ≤ 200 cells/mm^3^, low HGS, and low GS, and lower in asymptomatic and non-smoking individuals. The authors recommend investigating these factors in hospital and outpatient settings. The SARC—Calf proved to be more appropriate for screening sarcopenia risk in PLHIV.

## Highlights

• SARC—Calf is better than SARC-F in screening the risk of sarcopenia in PLHIV.
• Low CD4+ count, the presence of opportunistic infections, and AIDS status are associated with the risk of sarcopenia in PLHIV, regardless of the tool used.

## Introduction

Literature defines *sarcopenia* as a muscle disease characterized by generalized and progressive muscle strength and mass loss, with the ICD-10-CM diagnostic code.^1^ In People Living with Human Immunodeficiency Virus (PLHIV), sarcopenia development appears to occur earlier. Antiretroviral Therapy (ART) has increased the life expectancy of people infected with HIV. However, despite its significant benefits in treating the disease, the side effects of ART may cause damage to skeletal muscle. Some features associated with sarcopenia, such as low-grade chronic inflammation and prolonged use of ART, are also linked to PLHIV.^2^^,^^3^

A meta-analysis conducted by Oliveira et al. found a higher prevalence of sarcopenia in PLHIV compared to those without HIV. Patients with HIV were 6.1 times more likely to develop sarcopenia compared to those without it. Additionally, factors such as age, sex, body mass index, clinical characteristics, and sociodemographic variables were identified as risk factors for sarcopenia.^4^

Diagnosing sarcopenia can be challenging due to the high cost and limited availability of reference diagnostic methods in clinical practice.^5^ In this context, tools such as the SARC-F (Simple Questionnaire to Rapidly Diagnose Sarcopenia) and SARC—Calf have been proposed as viable methods for detecting sarcopenia risk. The SARC-F, developed by Malmstrom et al.,^6^ consists of five self-reported questions about muscle strength, walking ability, rising from a chair, climbing stairs, and falls. The SARC—Calf, proposed by Barbosa and Silva et al., includes the same five items as the SARC-F with the addition of Calf Circumference (CC).^7^

Studies suggest that the SARC—Calf tool may be promising due to its greater accuracy and practicality in identifying sarcopenia.^8^, ^9^, ^10^ However, no studies have compared the two tools or tested their agreement in PLHIV. To date, only one study has assessed sarcopenia risk using the SARC-F and its associated factors in PLHIV.^11^ This study included 202 PLHIV, mostly middle-aged (50.6–60.5 years). Only 5.9 % were found to be at risk of sarcopenia according to the SARC-F, and only gastrointestinal symptoms were associated with sarcopenia risk (OR = 1.06; 95 % CI 1.02‒1.09).

Given this background, the objectives of this study are: i) To assess sarcopenia risk and associated factors among PLHIV; ii) To compare the SARC-F and SARC—Calf tools for sarcopenia risk screening in PLHIV; iii) To evaluate the agreement between the SARC-F and SARC—Calf tools in screening for sarcopenia risk.

## Methods

### Study design, population, and location

This was a cross-sectional study with a non-probabilistic sample design and consecutive sampling, conducted from April 2023 to April 2024. The study was conducted according to the Strengthening the Reporting of Observational Studies in Epidemiology (STROBE) checklist. The authors invited People Living with HIV (PLHIV), who were undergoing ART, to participate in the study. These individuals were receiving care at the internal medicine clinic and infectious diseases outpatient clinic at the University Hospital of the Federal University of Grande Dourados (HU-UFGD), as well as the Specialized Care Service and Testing and Counseling Center (SAE/CTA) outpatient clinic.

HU-UFGD is a general hospital offering medium- to high-complexity care, including surgical, internal medicine, obstetric (specializing in high-risk pregnancies), pediatric, and mental health services. All services are part of the Unified Health System (SUS), focusing on patient care, teaching, research, and extension activities. According to the National Registry of Health Establishments (CNES), the hospital has 34 inpatient beds, 12 of which are allocated to infectious diseases. Outpatient services are provided in 25 medical offices, covering various medical specialties, including two offices dedicated exclusively to infectious diseases.

The SAE/CTA outpatient clinic is a reference center for the care and monitoring of people with Sexually Transmitted Infections (STIs) and PLHIV in the municipality of Dourados, state of Mato Grosso do Sul. The SAE/CTA offers testing, diagnosis, and treatment services for HIV, STIs, and viral hepatitis; pre- and post-exposure prophylaxis; immunization against hepatitis B and HPV; and distribution of both female and male condoms, among other services.

### Participant recruitment

At HU-UFGD, the authors identified participants through the appointment schedule, daily nursing census, and/or the hospital's electronic operating system. Each week, a trained researcher verified which patients met the eligibility criteria. Data was collected either at the bedside (inpatient care) or in a private room at the outpatient clinic.

For the SAE/CTA outpatient clinic, the clinic's multidisciplinary team, who referred potentially eligible patients to the researchers responsible for data collection, identified participants. Trained professionals, three times per week in the morning, conducted collection, and approached patients in a private room provided by the clinic.

### Inclusion and exclusion criteria

The authors considered patients aged ≥ 20-years, of both sexes, regardless of literacy, who had the cognitive ability to understand, perform the tests required in this study, and those PLHIV who were on ART. The authors excluded patients with total mobility impairment, cognitive difficulties and/or psychiatric disorders identified in their medical records, patients with edema or restrictions affecting hand strength assessments, and those who received their HIV diagnosis during their first hospitalization.

### Data collection procedures

#### Sociodemographic, lifestyle, and clinical variables

The authors obtained sociodemographic and lifestyle variables through interviews and extracted clinical data from patients’ medical records. The sociodemographic variables included age (in full years), marital status (single, married, widowed, or separated/divorced), employment status (binary: yes or no), self-declared race/color (white, black, brown, and yellow), later grouped into “white” and “non-white”, education level (< 4-years, 4‒8 years, > 8‒11 years, and > 11-years”), and economic level classified according to the Brazilian Economic Classification Criterion (ABEP).^12^

Lifestyle variables included alcohol consumption (categories: “non-drinker”, “occasional”, “weekly”, “daily”, and “former drinker”), smoking habits (“non-smoker/never smoked”, “yes, regular smoker” and “former smoker”), and Physical Activity Level (PAL) assessed through the short version of the International Physical Activity Questionnaire (IPAQ) for the Brazilian population.^13^ PAL was classified according to the World Health Organization (WHO) guidelines on physical activity and sedentary behavior^14^ and categorized as “Sufficient” for patients reporting 150 to 300 min of moderate physical activity or 75 to 150 min of intense physical activity per week, and “Insufficient” for those who did not meet these criteria.

Clinical variables included disease status (asymptomatic, symptomatic, and AIDS), CD4 T-cell count (cells/mm^3^) categorized as ≤ 200 cells/mm^3^ or > 200 cells/mm^3^, treatment duration (≤ 8-years and > 8-years), history of chronic diseases (binary: yes or no), and opportunistic infections (binary: yes or no).

### Sarcopenia risk assessment

Sarcopenia risk was the primary outcome of this study. The authors evaluated sarcopenia risk using the SARC-F tool proposed by Malmstrom ^6^ and the SARC—Calf, proposed by Barbosa and Silva et al.,^7^ both in their Portuguese versions. The SARC-F assesses five components: strength, assistance in walking, rising from a chair, climbing stairs, and falls. Each component is scored from 0 to 2 points, where 0 represents better functional capacity, and 2 represents worse functional capacity. A score ≥ 4 (out of a maximum of 10 points) indicates sarcopenia risk.

The SARC—Calf includes the same five items as the SARC-F but adds (CC) as a sixth component. The CC is scored 10 if the measurement is ≤ 34 cm in men and ≤ 33 cm in women, and 0 if the measurement exceeds these cutoffs. Thus, the SARC-Calf has a maximum score of 20 points, and individuals are classified as at risk for sarcopenia with scores ≥ 11, while those scoring < 11 are considered not at risk. The authors measured CC at the widest part of the calf using a flexible, non-elastic tape measure, following the criteria outlined by Lohman.^15^

### Sarcopenia components and diagnosis

#### Appendicular skeletal muscle mass index (ASMMI)

The authors determined muscle mass using the method proposed by Lee et al.^16^ The Appendicular Skeletal Muscle Mass Index (ASMMI) was calculated using the value obtained from Lee's equation divided by height squared. The value 0 was used for women and 1 for men. Values for race = -1.2 for Asians, 1.4 for African Americans, and 0 for Whites or Hispanics. The authors classified individuals with < 8.9 kg/m^2^ for men and < 6.4 kg/m^2^ for women as having low muscle mass, based on adaptations for the Brazilian population.^17^

ASMMI=(0.244×weight)+(7.8×height)+(6.6×sex)−(0.098×age)+(race−3.3).

**Equation 1:** Predictive equation for total skeletal muscle mass.

Note: ASMMI, Appendicular Skeletal Muscle Mass.

Muscle strength

The authors assessed Handgrip Strength (HGS, kg) using a SAEHAN® hydraulic hand dynamometer, model SH5001. Patients were first familiarized with the device and then examined while seated, with both arms bent at 90° at the elbow. They were instructed to grip the dynamometer and squeeze with maximum force. Each hand was measured three times, with a 1-minute interval between measurements, and the highest value for each hand was recorded. The authors defined low muscle strength as < 27 kg/f for men and < 16 kg/f for women.^1^

#### Physical performance

The authors measured physical performance using Gait Speed (GS, meters/seconds). Patients were instructed to walk at a usual pace for 4 m, repeated three times with a 1-minute interval between each trial. The fastest time was recorded. The authors defined low GS as ≤ 0.8 m/s.^1^

#### Sarcopenia

The authors used the algorithm proposed by the European Working Group on Sarcopenia in Older People (EWGSOP2)^1^ to diagnose sarcopenia. The authors categorized patients as “no sarcopenia”, “probable sarcopenia” (low muscle strength only), “confirmed sarcopenia” (low muscle strength and low muscle mass), or “severe sarcopenia” (low muscle strength, low muscle mass, and low GS). For this study, probable and severe sarcopenia were combined into a single category termed *sarcopenia*.

### Ethical considerations

This research adheres to the standards and guidelines of Good Clinical Practices as outlined in Resolution 466/2012. The Research Ethics Committee (REC) for human subjects under protocols 5.919.928 and 6.559.968 (amendment) and CAAE 67,159,123.9.0000.5160 approved the study. All patients who agreed to participate signed the Informed Consent Form (ICF). The researchers ensured the confidentiality and privacy of the data related to PLHIV, with database access restricted solely to the research team. Furthermore, to maintain participant anonymity, each individual was assigned a numerical code, safeguarding his or her identity within the database.

### Statistical analysis

Initially, the normality of the variables was tested using the Shapiro Wilk test (*p* > 0.05). Data were expressed as mean and standard deviation for continuous variables and percentages for categorical variables. Continuous variables were also presented as medians and Interquartile Ranges (IQR) when there was a non-normal distribution and high standard deviation. To compare mean scores between the SARC-F and SARC—Calf across sociodemographic, clinical variables, sarcopenia components, and sarcopenia classification, the authors applied the *t*-test for independent samples for variables with two categories and analysis of variance (ANOVA) for those with three or more categories. In cases where the assumption of homoscedasticity was violated, the authors applied Welch's correction and used Tukey's post-hoc test. Pearson's Chi-Square test (χ²) or Fisher's Exact Test was used to estimate associations between the SARC-F and SARC—Calf instruments and the categorical variables.

To assess the agreement between the instruments, the authors calculated the kappa coefficient (κ), considering values > 0.80 as almost perfect agreement, between 0.61 and 0.80 as substantial, between 0.41 and 0.60 as moderate, 0.21 to 0.40 as fair, 0.00 to 0.20 as slight, and < 0.00 as poor agreement.^18^ The authors analyzed data using IBM SPSS Statistics (v.22, SPSS, An IBM Company, Chicago, IL). The authors adopted a significance level of 5 % for all tests.

## Results

**Fig. 1:**
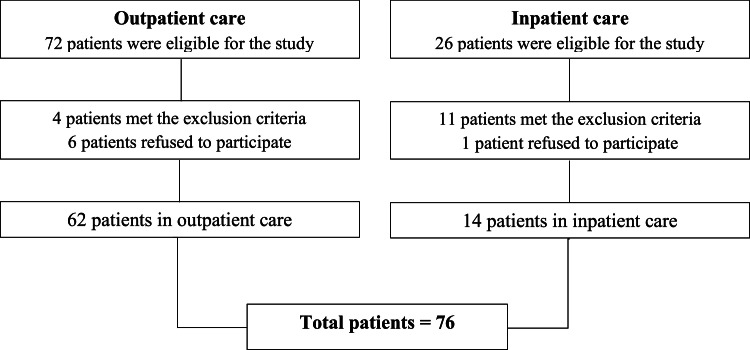
Flowchart of participant inclusion.

The authors included 76 patients in this study. The flowchart of participant inclusion is presented in Fig. 1. The sample consisted of 88.2 % adults (only 9 were elderly people, ≥ 60 years), and half of the individuals were male, with a mean age of 44.9 ± 12.7 years, with a maximum age of 71 years. Most patients self-identified as non-white (71.1 %), had no employment (68.4 %), were single (47.4 %), belonged to economic class C (56.6 %), and had 4 to 8 years of education (36.8 %) (Table 1). The mean ABEP score was 23.37 ± 8.19.

**Table 1:** Characterization of participants and mean scores of SARC-F and SARC—Calf instruments.

Variables	n	%	SARC-F	p*-*valueδ	SARC—Calf	p*-*δvalue
***Sociodemographic***						
**Sex**				0.375		0.075
Male	38	50.0	2.47 ± 2.46		8.26 ± 6.41	
Female	38	50.0	1.97 ± 2.42		5.66 ± 6.17	
**Age group**				0.565		0.081
Adult	67	88.2	2.16 ± 2.43		6.49 ± 6.35	
Older people (≥ 60-years)	9	11.8	2.67 ± 2.60		10.44 ± 5.88	
**Self-declared race/color**				0.752		0.459
White	22	28.9	2.36 ± 2.65		6.61 ± 6.13	
Non-white	54	71.1	2.17 ± 2.37		7.82 ± 7.05	
**Work activity**				0.212		0.029*
Absent	52	68.4	2.46 ± 2.45		8.04 ± 6.17	
Present	24	31.6	1.71 ± 2.39		4.63 ± 6.35	
**Marital status**				0.377		0.121
Single	36	47.4	2.67 ± 2.66		7.94 ± 6.26	
Married	27	35.5	1.81 ± 2.15		5.89 ± 6.48	
Widowed	3	3.9	3.00 ± 3.61		13.00 ± 3.61	
Separated/Divorced	10	13.2	1.50 ± 1.90		4.50 ± 6.08	
**Economic class**⁎⁎				0.004*		0.004*
Class A	1	1.3	5.00		5.00	
Class B	19	25.0	2.05 ± 2.46^b^		6.26 ± 6.34^b^	
Class C	43	56.6	1.63 ± 1.92^b^		5.58 ± 5.91^b^	
Classes D and E	13	17.1	4.23 ± 2.98^a^		12.69 ± 5.36^a^	
**Education level**				0.761		0.386
< 4-years	7	9.2	2.43 ± 3.16		8.14 ± 7.93	
4‒8-years	28	36.8	2.36 ± 2.64		7.00 ± 6.42	
8‒11-years	25	32.9	1.80 ± 2.18		5.40 ± 6.28	
> 11-years	16	21.1	2.56 ± 2.25		8.81 ± 5.76	
***Lifestyle habits***						
**Alcohol consumption**				0.647		0.312
Does not consume	37	48.7	2.49 ± 2.47		6.54 ± 6.59	
Occasionally	5	6.6	1.00 ± 2.24		3.00 ± 4.47	
Weekly	6	7.9	2.67 ± 2.07		11.00 ± 5.62	
Daily	18	23.7	2.22 ± 2.71		7.78 ± 6.62	
Ex-drinker	10	13.2	1.60 ± 2.22		6.60 ± 5.91	
**Smoking**				0.001*		0.009*
Does not smoke/never smoked	40	52.6	1.50 ± 1.9^b^		5.25 ± 5.56^b^	
Smokes regularly	18	23.7	2.00 ± 2.25^a^		7.00 ± 6.50^a^	
Ex-smoker	18	23.7	4.06 ± 2.84^b^		10.72 ± 6.70^a,b^	
**Physical activity level**				0.968		0.762
Insufficient	51	67.1	2.22 ± 2.39		7.12 ± 6.37	
Sufficient	25	32.9	2.24 ± 2.59		6.64 ± 6.53	
***Clinical***						
**Disease status**				<0.001*		<0.001*
Asymptomatic	35	46.1	1.03 ± 1.46^a^		3.89 ± 4.98^a^	
Symptomatic	13	17.1	2.54 ± 2.47^a,b^		8.69 ± 5.59^b^	
AIDS	28	36.8	3.37 ± 2.70^b^		10.00 ± 6.70^b^	
**Opportunistic infections**				0.001*		0.015*
Absent	47	61.8	1.53 ± 2.14		5.57 ± 5.97	
Present	29	38.2	3.34 ± 2.51		9.21 ± 6.50	
**Chronic disease history**				0.792		0.468
Absent	52	68.4	2.17 ± 2.30		6.60 ± 6.13	
Present	24	31.6	2.33 ± 2.75		7.75 ± 6.99	
**CD4 T-cell count**#				<0.001*		0.002*
≤ 200 cells/mm^3^	27	36.5	3.67 ± 2.70		9.96 ± 6.83	
> 200 cells/mm^3^	47	63.5	1.32 ± 1.72		4.94 ± 5.30	
**Treatment duration**				0.347		0.274
≤ 8-years	40	52.6	2.48 ± 2.44		7.73 ± 6.42	
> 8-years	36	47.4	1.94 ± 2.44		6.11 ± 6.32	
***Sarcopenia components***						
**Handgrip strength (HGS)**				<0.001*		0.001*
Adequate	63	82.9	1.68 ± 2.14		5.81 ± 6.00	
Low muscle strength	13	17.1	4.85 ± 2.12		12.54 ± 2.28	
**ASMI**				0.211		0.009*
Adequate	68	89.5	2.04 ± 2.26		6.31 ± 6.18	
Low muscle mass	8	10.5	3.75 ± 3.45		12.50 ± 5.68	
**Gait speed (GS)**				0.001*		<0.001*
Adequate	44	57.9	1.43 ± 1.83		4.84 ± 5.33	
Low gait speed	32	42.1	3.31 ± 2.75		9.88 ± 6.64	
**Sarcopenia**				<0.001*		0.001*
Absent	63	82.9	1.68 ± 2.14^a^		5.81 ± 6.00^a^	
Probable sarcopenia	9	11.8	4.44 ± 2.67^b^		11.11 ± 5.58^b^	
Confirmed and severe sarcopenia	4	5.3	5.75 ± 2.99^b^		15.75 ± 2.99^b^	

⁎ Statistical significance (*p* < 0.05) ^a,b,c^: same letters indicate statistical similarity.

⁎⁎ Average household income: *A* = *R*$21,826.74; *B* = *R*$5,755.23 to 10,361.48; C = R$1,965.87 to 3,276.76; DE = *R*$900.60; ASMMI: appendicular skeletal muscle mass index.

# CD4 T-cell count: *n* = 74 (2 *missing*). ^#^ Analysis of Variance (ANOVA).

δ *_t_*-test for independent samples or ANOVA and Tukey's test.

Regarding lifestyle habits, 48.7 % of the patients reported not consuming alcohol, 52.6 % were non-smokers or had never smoked, and 67.1 % had an insufficient level of physical activity.

As for clinical variables, 46.1 % of the patients were asymptomatic, 61.8 % had no opportunistic infections, and 68.4 % had no history of chronic diseases. The median CD4 T-cell count was 342.5 cells/mm^3^ (IQR: 115.5‒573.3 cells/mm^3^), and the median treatment duration was 95.5 months (IQR: 16.5‒180.0 months).

Most patients exhibited adequate HGS (82.9 %), ASMMI (89.5 %), and GS (57.9 %). The authors observed the absence of sarcopenia in most patients (82.9 %) (Table 1). The sample had a mean HGS of 29.0 ± 10.9 kg/f, ASMMI of 9.4 ± 1.7 kg/m^2^, and GS of 0.89 ± 0.32m/s.

The authors observed significant differences in the mean SARC-F scores regarding economic level (*p* = 0.004), smoking (*p* = 0.001), disease status (*p* < 0.001), opportunistic infections (*p* = 0.001), CD4 T-cell count (*p* < 0.001), sarcopenia construct, and its components HGS and GS, all at a significance level of 0.001 (Table 1).

For the SARC—Calf tool, the authors found significant differences in the mean scores of variables such as employment status (*p* = 0.029), economic class (*p* = 0.004), smoking (*p* = 0.009), disease status (*p* < 0.001), opportunistic infections (*p* = 0.015), CD4 T-cell count (*p* = 0.002), sarcopenia construct (*p* = 0.001), and all its components, including HGS (*p* = 0.001), ASMI (*p* = 0.009), and GS (*p* < 0.001) (Table 1).

These analyses indicated that patients in lower economic classes, smokers or former smokers, those diagnosed with AIDS, those with opportunistic infections, CD4 T cell count ≤ 200 cells/mm^3^, low HGS, low GS, and those with probable or confirmed sarcopenia exhibited higher scores on both the SARC-F and SARC—Calf. Additionally, individuals without employment and those with low muscle mass, as determined by ASMI, also had higher scores when assessed by the SARC—Calf (Table 1).

Agreement between the SARC-F and SARC—Calf tools was moderate (κ = 0.49; *p* < 0.001) (data not tabulated). Table 2 shows that the sarcopenia risk evaluated by the SARC-F was 27.6 %, while it was 36.8 % according to the SARC—Calf (*p* < 0.001).

**Table 2:** Sarcopenia risk using the SARC-F and SARC—Calf tools.

Sarcopenia risk	SARC-F		SARC—Calf		
	n	%	n	%	p-value^a^
No	55	72.4	48	63.2	<0.001
Yes	21	27.6	28	36.8	

a Chi-Square test.

Table 3 presents the relationship between sarcopenia risk, as assessed by the SARC-F and SARC—Calf, and sociodemographic, lifestyle, clinical variables, sarcopenia diagnosis, and its components.

**Table 3:** Relationship between the SARC-F and SARC—Calf tools and sociodemographic variables, lifestyle habits, clinical factors, sarcopenia components, and sarcopenia construct.

Variables	SARC-F			SARC—Calf		
	Not at risk	At risk	p#	Not at risk	At risk	p#
***Sociodemographic***						
**Sex**			0.305			0.234
Male	25 (65.8)	13 (34.2)		21 (55.3)	17 (44.7)	
Female	30 (78.9)	8 (21.1)		27 (71.1)	11 (28.9)	
**Age group**			0.701			0.276
Adult	49 (73.1)	18 (26.9)		44 (65.7)	23 (34.3)	
Older people	6 (66.7)	3 (33.3)		4 (44.4)	5 (55.6)	
**Self-declared race/color**			0.396			0.794
White	14 (63.6)	8 (36.4)		13 (59.1)	9 (40.9)	
Non-white	41 (75.9)	13 (24.1)		35 (64.8)	19 (35.2)	
**Work activity**			0.421			0.202
Absent	36 (69.2)	16 (30.8)		30 (57.7)	22 (42.3)	
Present	19 (79.2)	5 (20.8)		18 (75.0)	6 (25.0)	
**Marital status**			0.724			0.412
Single	24 (66.7)	12 (33.3)		21 (58.3)	15 (41.7)	
Married	21 (77.8)	6 (22.2)		18 (66.7)	9 (33.3)	
Widowed	2 (66.7)	1 (33.3)		1 (33.3)	2 (66.7)	
Separated/Divorced	8 (80.0)	2 (20.0)		8 (80.0)	2 (20.0)	
**Economic class**⁎⁎			0.007*			0.058
Class A	0 (0.0)	1 (100.0)		1 (100)	0 (0.0)	
Class B	15 (78.9)	4 (21.1)		13 (68.4)	6 (31.6)	
Class C	35 (81.4)	8 (18.6)		30 (69.8)	13 (30.2)	
Classes D and E	5 (38.5)	8 (61.5)		4 (30.8)	9 (69.2)	
**Education level**			0.491			0.872
< 4-years	4 (57.1)	3 (42.9)		4 (57.1)	3 (42.9)	
4‒8 years	21 (75.0)	7 (25.0)		18 (64.3)	10 (35.7)	
8‒11-years	20 (80.0)	5 (20.0)		17 (68.0)	8 (32.0)	
> 11-years	10 (62.5)	6 (37.5)		9 (56.3)	7 (43.8)	
***Lifestyle habits***						
**Alcohol consumption**			0.934			0.670
Does not consume	27 (73.0)	10 (27.0)		24 (64.9)	13 (35.1)	
Occasionally	4 (80.0)	1 (20.0)		5 (100.0)	0 (0.0)	
Weekly	4 (66.7)	2 (33.3)		1 (16.7)	5 (83.3)	
Daily	12 (66.7)	6 (33.3)		11 (61.1)	7 (38.9)	
Ex-drinker	8 (80.0)	2 (20.0)		7 (70.0)	3 (30.0)	
**Smoking**			0.006*			0.030*
Does not smoke/never smoked	34 (85.0)	6 (15.0)		30 (75.0)	10 (25.0)	
Smokes regularly	13 (72.2)	5 (55.6)		11 (61.1)	7 (38.9)	
Ex-smoker	8 (44.4)	10 (55.6)		7 (38.9)	11 (61.1)	
**Physical activity level**			0.786			0.801
Insufficient	36 (70.6)	15 (29.4)		33 (64.7)	18 (35.3)	
Sufficient	19 (76.0)	6 (24.0)		15 (60.0)	10 (40.0)	
***Clinical***						
**Disease status**			<0.001*			0.002*
Asymptomatic	33 (94.3)	2 (5.7)		29 (82.9)	6 (17.1)	
Symptomatic	8 (61.5)	5 (38.5)		8 (61.5)	5 (38.5)	
AIDS	14 (50.0)	14 (50.2)		11 (39.3)	17 (60.7)	
**Opportunistic infections**			0.002*			0.050
Absent	40 (85.1)	7 (14.9)		34 (72.3)	13 (27.7)	
Present	15 (51.7)	14 (48.3)		14 (48.3)	15 (51.7)	
**Chronic disease history**			0.789			0.614
Absent	37 (71.2)	15 (28.8)		34 (65.4)	18 (34.6)	
Present	18 (75.0)	6 (25.0)		14 (58.3)	10 (41.7)	
**CD4 T-cell count**#			0.001*			0.002*
≤ 200 cells/mm^3^	13 (48.1)	14 (51.9)		11 (40.7)	16 (59.3)	
> 200 cells/mm^3^	41 (87.2)	6 (12.8)		37 (78.7)	10 (21.3)	
**Treatment duration**			0.199			0.344
≤ 8-years	26 (65.0)	14 (35.0)		23 (57.5)	17 (42.5)	
> 8-years	29 (80.6)	7 (19.4)		25 (69.4)	11 (30.6)	
***Sarcopenia components***						
**Handgrip strength (HGS)**			<0.001*			0.002*
Adequate	52 (82.5)	11 (17.5)		45 (71.4)	18 (28.6)	
Low muscle strength	3 (23.1)	10 (76.9)		3 (23.1)	10 (76.9)	
**ASMI**			0.206			0.136
Adequate	51 (75.0)	17 (25.0)		45 (66.2)	23 (33.8)	
Low muscle mass	4 (50.0)	4 (50.0)		3 (37.5)	5 (62.5)	
**Gait speed (GS)**			0.002*			0.004*
Adequate	38 (86.4)	6 (13.6)		34 (77.3)	10 (22.7)	
Low gait speed	17 (53.1)	15 (46.9)		14 (43.8)	18 (56.3)	
**Sarcopenia**			<0.001*			0.002*
Absent	52 (82.5)	11 (17.5)		45 (71.4)	18 (28.6)	
Probable sarcopenia	2 (22.2)	7 (77.8)		3 (33.3)	6 (66.7)	
Confirmed and severe sarcopenia	1 (25.0)	3 (75.0)		‒	4 (100.0)	

⁎ Statistical significance (*p* < 0.05).

⁎⁎ Average household income: *A* = *R*$21,826.74; *B* = *R*$5,755.23 to 10,361.48; C = R$1,965.87 to 3,276.76; DE = *R*$900.60; ASMMI: appendicular skeletal muscle mass index.

# CD4 T-cell count: *n* = 74 (2 missing). ^#^Chi-Square test or Fisher's exact test.

The SARC-F showed a significant association with economic class, with patients in classes D and E having a higher proportion of sarcopenia risk (*p* = 0.007), regular smoking (*p* = 0.006), AIDS diagnosis (*p* < 0.001), presence of opportunistic infections (*p* = 0.002), CD4 T-cell count ≤ 200 cells/mm^3^, and low muscle strength (*p* < 0.001). Most patients with adequate GS (86.4 %) were not at risk for sarcopenia (*p* = 0.002), and the majority of those without sarcopenia (82.5 %) also had no risk of sarcopenia (*p* < 0.001).

For the SARC—Calf tool, a larger proportion of patients who were non-smokers or had never smoked were not at risk for sarcopenia (*p* = 0.030), and most asymptomatic patients were also free from sarcopenia risk (*p* = 0.002). Regarding CD4 T-cell count, most patients with counts > 200 cells/mm^3^ were not at risk for sarcopenia. Additionally, patients with low HGS (*p* = 0.002), low GS (*p* = 0.004), and confirmed or severe sarcopenia (*p* = 0.002) were at risk for sarcopenia, according to the SARC—Calf (Table 3).

## Discussion

In this study, the authors found that the prevalence of sarcopenia risk was higher with the SARC-Calf than with the SARC-F, with moderate agreement between them. This was expected since the SARC—Calf has better diagnostic accuracy measures (sensitivity and specificity). Adding (CC) enhances the tool's ability to detect actual cases of sarcopenia while minimizing false positives.^7^ Furthermore, the authors observed that factors associated with sarcopenia risk included the absence of employment, low family income (average per capita income of 900.60 reais),^12^ former smokers, Advanced Disease Stage (AIDS), opportunistic infections, low CD4 T-cell count, low muscle strength and mass, slow gait, and probable or confirmed sarcopenia. The factors related to sarcopenia risk were similar for both instruments.

The EWGSOP2 guidelines currently recommend the SARC-F to identify sarcopenia risk in the general older population. However, studies indicate that while SARC-F has high specificity, its sensitivity is lower.^19^, ^20^, ^21^ The lower sensitivity is likely because it does not require information on muscle mass, making the SARC—Calf a more promising tool.^22^ Nevertheless, no studies have yet evaluated the agreement between these instruments in PLHIV.

In the present study, the agreement between SARC-F and SARC—Calf was moderate (*k* = 0.49). A study with hemodialysis patients found sarcopenia risk at 23 % using SARC-F and 40 % with SARC—Calf, with moderate agreement between both tools in identifying probable sarcopenia due to low muscle strength (FPM). Both tools showed a moderate kappa coefficient with slowness and probable sarcopenia due to low handgrip strength. Sensitivity for probable sarcopenia was higher with the SARC—Calf than with the SARC-F (70 % vs. 30 %). These authors concluded that the SARC—Calf is more strongly associated with probable sarcopenia and has greater sensitivity for detecting it because it incorporates muscle mass measurement.^23^

Regardless of the instrument used, the authors observed higher scores in patients with lower economic status and lower scores in those with higher economic levels. In this study, patients in economic class DE had a higher proportion of sarcopenia risk, according to the SARC-F. Several socioeconomic factors, such as limited access to nutritious food and healthcare, increase vulnerability to muscle mass loss and muscle weakness, components of sarcopenia.^24^

Patients without employment had higher scores on the SARC-F than those with jobs. Most patients in this study reported being unemployed, which may explain this finding. In Brazil, PLHIV can retire due to work incapacity.^25^ Reduced physical activity and increased sedentary behavior during this phase are significant risk factors for sarcopenia development.^1^ Physical inactivity contributes to decreased muscle protein synthesis and increased degradation, resulting in muscle strength and mass loss.^26^

Former smokers had higher sarcopenia risk scores on both instruments. Supporting this, the authors also observed that most patients who reported never smoking or no longer smoking did not have sarcopenia risk. Gao et al.^27^ found in a systematic review and meta-analysis that smoking was associated with a higher risk of sarcopenia (OR = 1.20, 95 % CI: 1.10–1.21). This is because smoking triggers inflammation and oxidative stress, which impair protein synthesis and negatively affect muscle health.^27^

Regarding clinical variables, the authors found that regardless of the tool used, patients with AIDS, opportunistic infections, and low CD4 T-cell count (≤ 200 cells/mm^3^) had higher sarcopenia risk scores. Additionally, asymptomatic patients, those without opportunistic infections, and those with CD4 T-cell count > 200 cells/mm^3^ were not at risk for sarcopenia.

Disease progression to AIDS is closely linked to inadequate treatment. Low adherence to ART leads to increased viral load and decreased CD4 T-cell count, accelerating HIV progression to AIDS. The immune system weakens in this phase, allowing opportunistic infections to arise.^28^^,^^29^ These factors may make individuals more susceptible to sarcopenia, though this relationship is rarely discussed in the literature.^3^^,^^30^

Although there are limited studies explaining the association between sarcopenia risk and low CD4 T-cell count, systemic inflammation and increased immune activation caused by disease progression contribute to muscle mass loss and sarcopenia development.^31^ Guimarães et al.^32^ concluded in their systematic review and meta-analysis that PLHIV has lower muscle mass than people without HIV, and this difference may be influenced by CD4 T-cell count and ART use.^32^

Scores were higher in patients with low muscle strength, low physical performance, and probable or confirmed severe sarcopenia. Moreover, patients with adequate muscle strength, gait speed, and absence of sarcopenia did not show sarcopenia risk. The adverse effects of ART have been identified as significant risk factors for sarcopenia in PLHIV.^3^ Fat accumulation in skeletal muscle, typical in PLHIV, activates pro-inflammatory pathways, altering skeletal muscle quality and quantity.^11^ Previous studies have also shown lower skeletal muscle density in PLHIV due to increased fatty acid infiltration in the skeletal muscle.^33^^,^^34^

In terms of muscle mass determined by the ASMI, patients with low muscle mass only had higher scores when assessed by the SARC—Calf, which was expected as low muscle mass is a key factor in sarcopenia risk.^35^ Furthermore, these findings support the evidence that the SARC—Calf is more sensitive to muscle mass than the SARC-F,^8^ even though the authors used a predictive equation to determine muscle mass. The authors emphasize that in contexts where other muscle mass assessment methods are unavailable, the predictive equation for appendicular muscle mass seems a viable alternative, as recommended by experts in Brazil.^36^

The authors need to highlight this study's limitations and strengths. As it is a cross-sectional study, the authors cannot infer causality. The authors used a predictive equation to estimate ASMI, but we recognize that more robust methods exist to determine skeletal muscle mass. These methods, however, are costly and often unavailable for research in public health services. In addition, a low number of individuals enrolled in the study. However, the authors conducted the study with rigorous data collection and analysis methods. To date, there is no study using SARC—Calf toll in PLHIV and this is the first study to compare the SARC-F and SARC—Calf tools with risk factors in PLHIV.

## Conclusion

The authors observed sarcopenia risk in approximately one-third of the present sample when using the SARC—Calf tool, while the SARC-F identified about one-quarter of the sample at risk. Both instruments were associated with variables such as economic status, smoking habits, disease stage, opportunistic infections, CD4 T-cell count, sarcopenia construct, handgrip strength, and gait speed. The authors encourage investigating these variables in clinical practice.

Despite the similarities between the tools regarding associated variables, the authors recommend using the SARC—Calf to screen sarcopenia risk in PLHIV, especially because it allows for assessing muscle mass. This simple, quick, cost-effective, and non-invasive tool contributes to more reliable sarcopenia screening in PLHIV in public health settings and clinical practices across various healthcare environments.

## CRediT authorship contribution statement

**Lara Cristina Vieira:** Investigation, Formal analysis, Writing – original draft, Writing – review & editing. **Jaine Alves Ximenez:** Investigation, Data curation, Formal analysis, Writing – original draft, Writing – review & editing. **Maria Claudia Bernardes Spexoto:** Conceptualization, Methodology, Formal analysis, Data curation, Validation, Writing – review & editing, Visualization, Supervision, Project administration.

## Conflicts of interest

The authors declare no conflicts of interest.
